# The Potential of Matrine in the Treatment of Breast Cancer: A Review

**DOI:** 10.3390/biomedicines13061355

**Published:** 2025-05-31

**Authors:** Yumin Yang, Yufeng Li, Shanshan Liao, Pan Gao, Jie Tian, Cheng Fu, Xuhua Qin, Shenrui Jin

**Affiliations:** 1 College of Pharmacy, Chengdu University of Traditional Chinese Medicine, Chengdu 611137, China; yymshmily@163.com (Y.Y.); 15391537265@163.com (Y.L.); liaoshanshan@stu.cdutcm.edu.cn (S.L.); lbyisgp427@126.com (P.G.); jaytian@stu.cdutcm.cn (J.T.); 17628332768@163.com (C.F.); 2 School of Basic Medical Sciences, Chengdu University of Traditional Chinese Medicine, Chengdu 611137, China

**Keywords:** matrine, breast cancer, anti-tumor, mechanism, traditional Chinese medicine monomer

## Abstract

Breast cancer ranks as the fifth-most-prevalent malignancy worldwide, characterized by high heterogeneity and multifactorial etiology across molecular subtypes. Despite advancements in conventional therapies, including surgery and chemotherapy, persistent challenges such as treatment-related adverse effects and acquired drug resistance necessitate alternative therapeutic strategies. Matrine, a naturally occurring alkaloid derived from Sophora flavescens, has demonstrated significant anticancer potential through multiple mechanisms. Experimental evidence indicates that matrine exerts inhibitory effects on tumor cell proliferation, promotes apoptosis, and attenuates metastatic progression via modulation of critical signaling pathways, particularly PI3K/Akt, JAK/STAT, NF-κB, MAPK/ERK, and Wnt/β-catenin. This review systematically examines subtype-specific responses to matrine treatment, highlighting its potential utility in precision oncology for distinct breast cancer classifications. Furthermore, we evaluate matrine’s capacity to synergize with standard chemotherapeutic regimens, potentially overcoming drug resistance while reducing required dosages. By integrating current preclinical and clinical findings, this analysis provides new perspectives on matrine’s therapeutic applications and underscores the imperative for translational studies to establish optimized treatment protocols for clinical implementation.

## 1. Introduction

Breast cancer has emerged as a leading global health challenge, representing one of the most prevalent and aggressive malignancies. According to the International Agency for Research on Cancer (IARC) 2020 report, it is the fifth-leading cause of cancer-related mortality worldwide, and its incidence continues to rise [1]. This epidemiological trend has intensified the search for safer and more effective therapeutic agents, positioning natural compounds as promising candidates for oncological research. Among these, matrine (MT), a tetracyclic quinolizidine alkaloid, has garnered significant scientific interest due to its multifaceted pharmacological profile. Isolated primarily from *Sophora flavescens* Aiton (*S. flavescens*), with additional sources including *Sophora alopecuroides* L. (*S. alopecuroides*), and *Euchresta japonica* Benth. ex Oliv. (*E. japonica*) [2]. This compound exhibits a remarkable spectrum of bioactivities spanning anti-neoplastic, anti-inflammatory, antimicrobial, antiviral, and neuroprotective properties [3,4,5,6,7,8,9,10,11,12,13]. Among these diverse pharmacological activities, its anti-cancer efficacy, particularly in breast cancer, has been widely explored, with promising results observed in both preclinical and clinical settings. However, despite these advances, a comprehensive analysis of its mechanisms of action and therapeutic potential, specifically in breast cancer, remains scarce.

**Figure 1:**
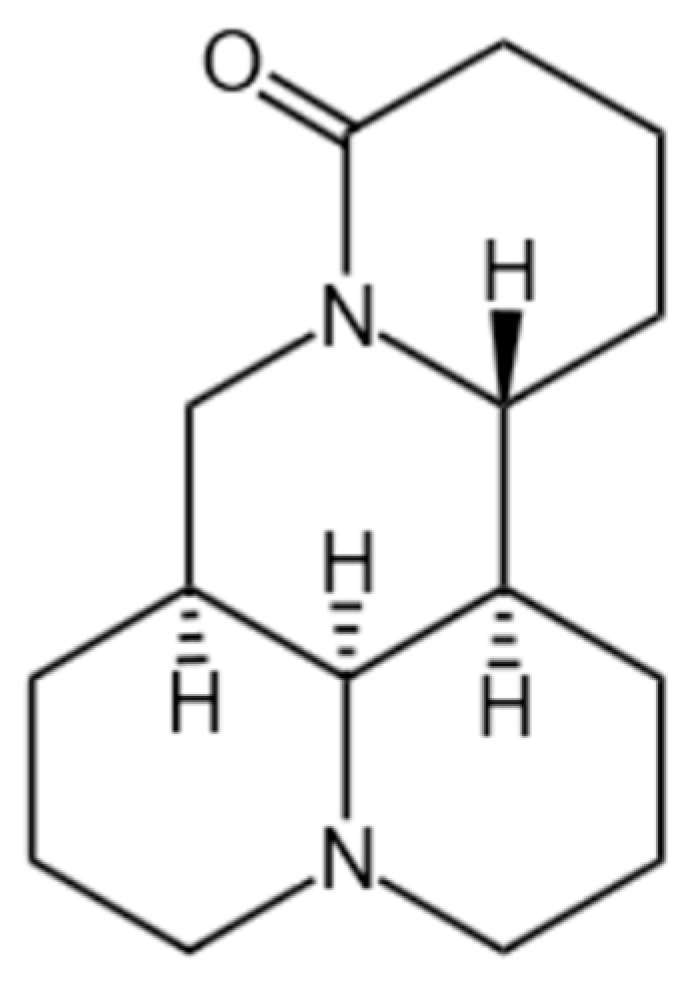
Structural formula of MT.

**Figure 2:**
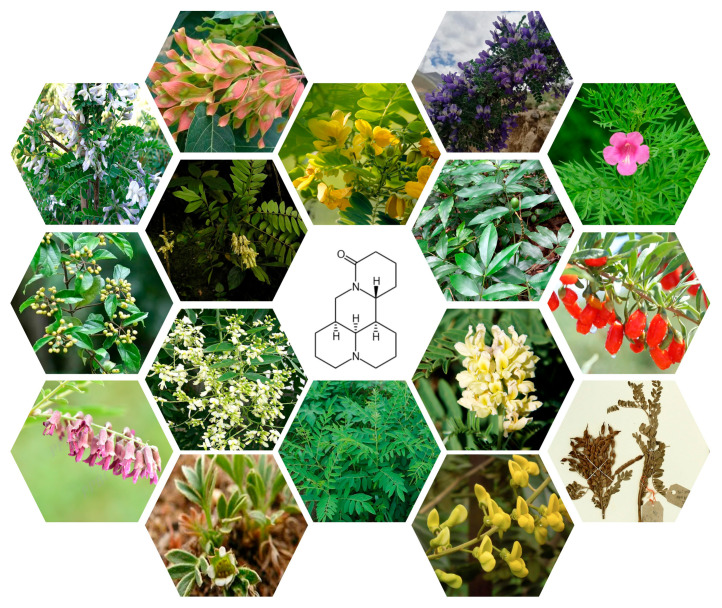
Pharmacological activity diagram of MT.

MT, a tetracyclic quinolizidine alkaloid first isolated from *S. flavescens* in 1958, has been identified as a key bioactive component in traditional Chinese medicine (TCM) [3,14]. The chemical formula is C_15_H_24_N_2_O with a molecular weight of 248.36, as depicted in Figure 1. It is found in various natural plants. Detailed information on its botanical sources is provided in Table 1 and Figure 2 [15,16,17,18,19,20,21,22,23,24,25,26,27,28]. Historical records from Shen Nong Ben Cao Jing (circa 200 CE) described *S. flavescens* in treating tumor-like masses and toxin-related disorders, laying the empirical foundation for modern pharmacological investigations [29]. Subsequent experimental studies validated its antitumor properties, particularly through MT-mediated inhibition of tumor cell proliferation [30]. Several literature studies have shown that MT can exert anti-cancer activity through multiple signal pathways, and it has pharmacological activity against breast cancer, lung cancer, liver cancer, blood cancer, gastric cancer, pancreatic cancer, and so on [31,32,33]. Clinical applications were advanced by Professor Wang Xixing’s successful use of *S. flavescens* in gynecological tumor management [34]. Additionally, the Compound Kushen Injection (CKI) demonstrated efficacy in alleviating cancer-related pain and hemorrhage [35,36]. These historical and clinical observations collectively support MT’s potential in suppressing tumor growth, inducing apoptosis, and inhibiting metastasis [31,32,33].

In breast cancer, MT exhibits multifaceted antitumor effects via modulation of critical signaling pathways. Mechanistic studies reveal its dual regulatory action on the PI3K/Akt/mTOR and MAPK/ERK axes, coupled with suppression of JAK/STAT signaling. Furthermore, MT activates apoptosis through upregulation of Bax and caspase-3 while simultaneously promoting autophagy via LC3-II conversion and Beclin-1 activation. Its antimetastatic properties are mediated through the downregulation of MMP-9 and VEGF, effectively inhibiting angiogenesis and epithelial–mesenchymal transition (EMT). Preclinical models consistently demonstrate MT’s capacity to impede breast cancer progression across multiple molecular subtypes.

**Figure 3:**
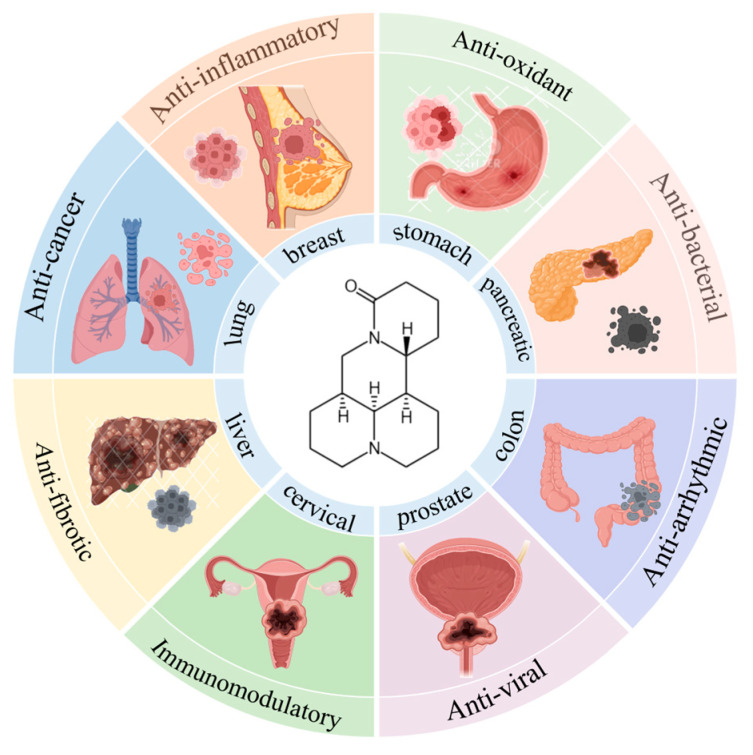
Graph of activity of MT.

Beyond breast cancer, MT displays broad-spectrum anticancer activity against lung, liver, and pancreatic malignancies through tissue-specific mechanisms [37,38,39,40,41,42,43,44,45,46,47,48,49] (as shown in Figure 3). The ability of MT to modulate oxidative stress and reduce inflammation further contributes to its potential as a multi-target therapy for breast cancer. Its anti-inflammatory effects are particularly noteworthy, as inflammation is a known contributor to cancer progression and metastasis. Additionally, MT has demonstrated neuroprotective effects, making it a promising candidate for patients experiencing cancer-related pain, or those undergoing chemotherapy.

While substantial evidence supports the therapeutic potential of MT in breast cancer treatment, further research is needed to fully elucidate its molecular mechanisms of action, optimize dosing strategies, and assess its safety profile in clinical settings. This review aims to provide a thorough overview of MT’s anti-breast cancer properties. This review fills the gap in the existing literature on MT treatment of breast cancer.

## 2. Materials and Methods

A comprehensive and systematic literature search was conducted across eight major biomedical databases, including ScienceDirect, PubMed, CNKI, and VIP, covering studies published from January 1996 to May 2025. The search strategy utilized Medical Subject Headings (MeSH) terms in combination with Boolean operators, as follows: (“matrine” OR “Sophora flavescens”) AND (“breast neoplasms” [MeSH] OR “breast cancer” OR “mammary carcinoma”) AND (“in vivo” OR “in vitro”).

Inclusion criteria required original experimental studies evaluating the antitumor effects of matrine using in vitro and/or in vivo models. Review articles, commentaries, and studies lacking mechanistic data were excluded. In total, 2137 records were initially retrieved. After independent screening by two reviewers, a final set of 59 eligible studies was selected for analysis.

The following parameters were extracted and analyzed: Cellular-level outcomes, including IC_50_ values, apoptosis rates (via Annexin V/PI assays), and migration inhibition (via scratch/wound healing assays), as well as in vivo pharmacodynamic indicators, such as tumor volume reduction (%) and tumor weight changes in xenograft mouse models, and molecular mechanisms.

Studies that lacked both in vitro and in vivo experimental data on matrine in the context of breast cancer were excluded from the final synthesis. Importantly, no registered clinical trials evaluating the efficacy of matrine in breast cancer patients were identified during the search. Data synthesis and quantitative analyses were performed using STATA version 17, where applicable, to conduct meta-analyses and evaluate pooled effect sizes.

## 3. Results

MT exhibits dose-dependent antiproliferative effects across diverse breast cancer subtypes, as evidenced by in vitro and in vivo studies summarized in Table 2 [50,51,52,53,54,55,56,57,58,59,60,61,62,63,64,65,66,67,68,69,70,71,72,73,74,75,76,77,78,79,80,81,82,83,84,85,86,87,88,89,90,91,92,93,94,95,96,97,98,99] and Table 3 [65,83,88,94,100,101,102,103,104]. This study aims to characterize the pharmacological profile of MT against breast cancer and to explore potential subtype-specific differences in its mechanisms of action. The ultimate objective is to support the development of personalized, subtype-targeted therapeutic strategies for breast cancer treatment.

### 3.1. In Vitro Experimental Study of MT on Breast Cancer Cells

A systematic review of the literature was performed to assess the in vitro anticancer properties of MT in breast cancer. The included studies investigated MT’s effects in a wide array of breast cancer cell lines, including triple-negative breast cancer (TNBC) cells such as MDA-MB-231, BT474, MDA-MB-468, HCC-1806, and BT20; ER+/PR+ breast cancer cell lines such as MCF-7, T47-D, and Bcap-37; HER2+ cell lines such as SK-BR-3; and the murine breast cancer cell line 4T1. Assays used to evaluate cell viability and proliferation included MTT, CCK-8, crystal violet (CV), propidium iodide (PI), and SRB. Apoptosis and cell cycle arrest were assessed using methods such as flow cytometry, TUNEL immunofluorescence, and quantitative image analysis, as shown in Table 2.

Experimental results consistently demonstrated that MT exerts significant cytotoxic effects on multiple breast cancer cell types by inhibiting proliferation, inducing cell cycle arrest, and promoting apoptosis and autophagy. Furthermore, MT was found to suppress cancer cell migration and invasion, highlighting its strong potential as an anti-breast cancer agent.

In TNBC cell lines (e.g., MDA-MB-231, MDA-MB-468, BT474, and BT20), MT inhibited cell proliferation and induced apoptosis in a concentration- and time-dependent manner. For instance, Shao et al. [82] reported that treatment with 3 mg/mL MT for 48 h resulted in an approximately 90% apoptosis rate in MDA-MB-231 cells as measured by the MTT assay, indicating potent cytotoxic effects at relatively low concentrations. Mechanistically, the anti-TNBC effects of MT were mediated via modulation of the PI3K/Akt, MAPK/ERK, and NF-κB-signaling pathways. MT downregulated the expression of N-cadherin, vimentin, Bcl-2, IκB, HN1, VEGF, and CD31 while decreasing the Bcl-2/Bax ratio. Additionally, mRNA levels of MMP-9, MMP-2, EGF, and VEGFR1 were reduced, whereas the expression of E-cadherin, AKT, ERK1/2, and p38 was elevated. Caspase-3 activation was increased, and LC3-II expression was enhanced, further supporting MT’s pro-apoptotic and pro-autophagic actions in TNBC cells.

In ER+/PR+ breast cancer cell lines (MCF-7, T47-D, and Bcap-37), MT exhibited strong inhibitory effects, with reported IC_50_ values ranging from 0.3 to 15.8 mg/mL. MCF-7 cells demonstrated the highest sensitivity, with IC_50_ values between 0.7–0.9 mg/mL. The underlying mechanism involved mitochondrial membrane potential disruption and regulation of apoptosis-related gene expression. MT treatment led to the downregulation of Bcl-2 and NF-κB p65 while upregulating Bax, p53, caspase-3, PARP, Beclin-1, LC3b-II, and GSK-3β. Additionally, MT inhibited the IL-6/JAK/STAT3-signaling pathway, as evidenced by decreased expression of IL-6, JAK1, p-JAK1, STAT3, and p-STAT3. It also downregulated Wnt/β-catenin signaling and VEGF expression. Specific molecular alterations included decreased levels of IKKβ, cyclin D1, c-Myc, PI3K, Akt, P-gp, p62, CD133, and KLF4, while the expression of p27/Kip1 and let-7b was increased. These changes promoted cytochrome c release and caspase-3 activation, culminating in apoptosis. Furthermore, MT induced cell cycle arrest in MCF-7 cells, as shown by increased accumulation in the G0/G1 or G1 phases, with corresponding reductions in S and G2/M phase populations. MT also inhibited telomerase activity, contributing to reduced cell proliferation and enhanced apoptotic activity.

In HER2+ breast cancer cell lines, the anticancer effects of MT were associated with inhibition of miR-21 expression and upregulation of PTEN, resulting in suppressed cell proliferation. In the murine 4T1 cell line, treatment with 0.4 mg/mL MT for 48 h resulted in an apoptosis rate of 17.32 ± 3.09% [84], indicating moderate cytotoxic and pro-apoptotic effects. Further investigations revealed that MT may exert its antitumor effect in this model through the regulation of ANXA3 protein expression.

### 3.2. Analysis of In Vivo Experimental Studies of MT Against Breast Cancer Cell Lines

A systematic review of in vivo studies evaluating the anti-tumor efficacy of MT in breast cancer models was conducted. These studies employed tumor-bearing animal models established using various breast cancer cell lines, including MCF-7, TM40D, 4T1, MDA-MB-231, MDA-MB-451, Walker 256, and MA737. The therapeutic effects of MT on tumor progression were assessed primarily by measuring tumor volume and weight, as detailed in Table 3. Results from these in vivo studies demonstrated that MT significantly inhibited tumor growth and delayed tumor formation in treated animals. Notably, MT also exhibited preventive effects against early-stage mammary tumor development, suggesting its potential role in both therapeutic and prophylactic settings. Histopathological analyses of tumor tissues from MT-treated groups revealed several notable changes: a reduction in mitotic index, decreased invasiveness of tumor cells, and a marked suppression of angiogenesis and apoptosis dysregulation. Following the establishment of tumors, MT administration led to a significant reduction in tumor growth rate, final tumor weight, and the number of pulmonary metastatic nodules, indicating its potential to inhibit both primary tumor expansion and metastatic spread.

Collectively, these in vivo findings corroborate the potent anti-tumor properties of MT observed in in vitro settings and underscore its therapeutic promise for breast cancer. However, despite these encouraging outcomes, the current body of in vivo research remains relatively limited in scope and depth. Therefore, future studies should prioritize comprehensive in vivo investigations, including dose-response evaluations, long-term toxicity assessments, and comparative studies across different breast cancer subtypes, to further clarify MT’s therapeutic potential and facilitate its clinical translation.

## 4. Molecular Mechanisms of MT in Breast Cancer

In order to further study the mechanism of the effects of MT on breast cancer, this paper summarizes and analyzes the results of the above experimental studies and describes the anti-breast cancer effects of MT through multi-targets and multi-signaling pathways, which are described as follows.

### 4.1. Cytotoxic Effects of MT in Breast Cancer Cells

The cytotoxic impact of MT on various breast cancer cell lines has been extensively studied, showcasing a robust reduction in cell viability. In a study conducted by Shao et al., it was observed that MT treatment at a concentration of 3 mg/mL for 48 h led to a significant reduction in cancer cell viability, with reductions ranging from 76.4% to 84.5% across MCF-7, BT-474, and MDA-MB-231 cells [82]. This suggests a potent cytotoxic effect of MT against these breast cancer cells. Zhang’s research also reinforced this finding, revealing concentration- and time-dependent inhibitory effects on MCF-7 cell proliferation, with the optimal concentration identified as 2.5 mg/mL [105]. Additionally, Xiao et al. demonstrated that MT suppressed the proliferation of SK-BR-3 cells, with molecular investigations indicating a reduction in miR-21 expression and upregulation of PTEN, which may contribute to the anti-proliferative action of MT [68]. Furthermore, MT was shown to downregulate ANXA3 protein expression in 4T1 cells, leading to a reduction in cell proliferation [67]. In a related study by Zhang et al., the inhibition of AKT phosphorylation and the enhancement of PTEN expression were observed following MT treatment, providing additional evidence for its cytotoxic potential against cancer cells [106]. Moreover, Du et al. observed notable morphological alterations such as cell contraction, membrane blistering, and partial detachment in MCF-7 cells after 24 h of MT exposure, indicative of its cytotoxicity in breast cancer cells [81].

### 4.2. Induction of Cancer Cell Cycle Arrest by MT

**Figure 4:**
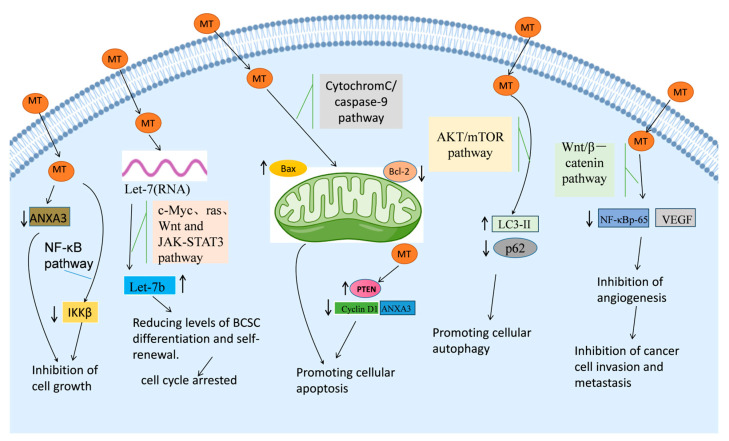
Anti-cancer mechanism of MT against breast cancer.

The cell cycle, a fundamental process regulating cell division and growth, consists of distinct phases: G0 (quiescent), G1 (early DNA synthesis), S (DNA synthesis), G2 (late DNA synthesis), and M (mitosis). The progression through key checkpoints, particularly G0/S and G2/M, is essential for successful cell division and homeostasis [57]. In vitro experiments conducted by Sui Hui et al. revealed that MT significantly altered the dynamics of the cell cycle in breast cancer cells. MT induced a marked increase in the proportion of cells arrested in the G0/G1 phase, accompanied by a decrease in the S phase population, thereby halting the cell cycle and triggering apoptosis in cancer cells [57]. This cell cycle arrest was likely mediated through the modulation of cell cycle-related proteins, which play a pivotal role in regulating tumor progression [107]. Li et al. found that MT treatment increased the expression of Let-7, a miRNA known for its tumor suppressive properties, particularly Let-7b, which is involved in the regulation of c-Myc, ras, and JAK-STAT3 pathways [79]. The upregulation of Let-7b led to a blockade of the Wnt-signaling pathway and inhibited the differentiation and self-renewal of breast cancer stem cells (BCSCs), further contributing to the overall anticancer effects of MT. Additionally, MT also inhibited key signaling pathways such as c-Myc, ras, Wnt, and JAK-STAT3 through Let-7 modulation, which likely played a central role in disrupting the cancer cell cycle and mediating its anticancer effects, as summarized in Figure 4.

### 4.3. Induction of Apoptosis in Cancer Cells by MT

Apoptosis, a genetically programmed form of cell death, is essential for maintaining cellular homeostasis and eliminating damaged cells. Dysregulated apoptosis is a hallmark of cancer, allowing for the evasion of cell death and tumor progression [108,109,110]. In breast cancer, apoptosis evasion is largely mediated by an imbalance between pro-apoptotic and anti-apoptotic proteins, notably Bax and Bcl-2. Bax induces mitochondrial outer membrane permeabilization (MOMP), leading to the release of cytochrome c and subsequent activation of caspases, which drive apoptosis [42]. MT has been shown to trigger apoptotic cell death in MCF-7 cells by altering the mitochondrial membrane potential (MMP), resulting in structural changes that facilitate the release of apoptotic factors. This cascade of events includes the regulation of Bcl-2 and Bax proteins, which ultimately culminates in the promotion of apoptosis [90,111]. Additionally, MT has been demonstrated to inhibit the PI3K/Akt pathway by suppressing mTOR activity, thus sensitizing cells to apoptosis. In Bcap-37 cells, treatment with 2 mg/mL MT led to a 19.58% apoptosis rate after 24 h of treatment, highlighting the dose-dependent pro-apoptotic effect of MT [66]. Furthermore, MT upregulated PTEN expression and inhibited miR-21, further confirming its ability to promote apoptosis in SK-BR-3 cells through the suppression of cell-proliferation pathways [68]. The induction of apoptosis by MT in MCF-7 cells was also evidenced by an increase in apoptotic vesicle formation after 48 h of treatment, with a significant increase in the apoptosis rate to 49.12 ± 3.79%. This suggests that apoptosis is a key mechanism by which MT exerts its anticancer effects in vivo.

### 4.4. Induction of Autophagy in Cancer Cells by MT

Cell death encompasses three distinct pathways: apoptosis, autophagy, and necrosis [112,113]. Autophagy, a process by which cells degrade and recycle their damaged components, is another form of programmed cell death and has gained attention as a therapeutic target in cancer treatment [114,115]. MT has been found to induce autophagy in MCF-7 cells through the modulation of key signaling pathways. Specifically, Du et al. observed an increase in LC3-II, an autophagy marker, alongside a reduction in p62, a protein involved in the autophagic degradation process [81]. Furthermore, MT treatment led to a decrease in the phosphorylation levels of AKT and mTOR in MCF-7 cells, signaling the activation of autophagy. Ren Lili et al. demonstrated that MT inhibited the phosphorylation of mTOR, suppressing the downstream activation of p70S6k and eIF4E, key regulators of protein synthesis and cell growth, thereby promoting autophagy in Bcap-37 cells [111]. These findings suggest that MT can modulate the AKT/mTOR pathway to enhance autophagic flux and promote autophagy in cancer cells, contributing to its anti-cancer effects, as depicted in Figure 4.

### 4.5. Inhibition of Angiogenesis by MT

Angiogenesis, the formation of new blood vessels, is crucial for tumor growth and metastasis [116]. In breast cancer, tumor angiogenesis is primarily regulated by the VEGF-signaling pathway, which is activated by the hypoxic conditions in tumors. MT has been shown to suppress angiogenesis by inhibiting the Wnt/β-catenin-signaling pathway and reducing VEGF expression, thereby limiting tumor blood supply and growth [83]. VEGF is a potent pro-angiogenic factor that promotes the formation of new blood vessels by binding to VEGFR1 and VEGFR2 on endothelial cells [117]. Li et al. demonstrated that MT treatment effectively downregulated VEGF protein expression, consequently inhibiting breast cancer cell proliferation and tumor angiogenesis [56]. Additionally, the experiments by Yu et al. [98] demonstrated that MT was found to reduce NF-κB p65 protein levels and lower the expression of MMP-9, MMP-2, EGF, and VEGFR1 mRNA in MDA-MB-231 cells, highlighting its anti-angiogenic potential in breast cancer treatment.

### 4.6. Inhibition of Cancer Cell Metastasis

Cancer metastasis, the spread of tumor cells to distant organs, is a significant cause of breast cancer mortality [118,119]. One of the key mechanisms involved in metastasis is epithelial-to-mesenchymal transition (EMT), characterized by the downregulation of e-cadherin and upregulation of N-cadherin, leading to enhanced cell motility and invasiveness. In a study by Ren et al., MT treatment led to an upregulation of e-cadherin and downregulation of N-cadherin in MDA-MB-231 and MCF-7 cells, effectively inhibiting EMT and reducing the metastatic potential of these cells [86]. By modulating EMT-related markers, MT may disrupt the metastatic cascade, offering a promising therapeutic strategy to prevent the spread of breast cancer.

### 4.7. Regulation of Immune Function by MT

Breast cancer progression is closely associated with immune dysfunction, particularly in the modulation of cytokine levels [120,121,122,123]. Significant changes in serum cytokine levels were observed in experiments with breast cancer cells in rats. The levels of IL-2 and IFN-γ exhibited a significant reduction, while the levels of IL-6, IL-10, and TGF-β showed an increase in the model group as compared to the control group. MT showed a remarkable ability to reverse these changes, restoring the balance of inflammatory factors and effectively preventing the growth of mammary tumors [101]. MT also modulated T-lymphocyte subpopulations, increasing CD8+ levels while decreasing CD3+ and CD4+ levels, indicating a shift towards a more effective anti-tumor immune response. Additionally, MT treatment resulted in reduced serum immunoglobulin levels (IgG, IgM, and IgA), suggesting its ability to restore normal immune function and prevent breast tumor growth.

### 4.8. Reversing Drug Resistance in Cancer Cells by MT

Multidrug resistance (MDR) is a major challenge in cancer treatment, often associated with the overexpression of drug-resistant genes such as MDR1, which encodes P-glycoprotein (P-gp). This protein pumps chemotherapeutic agents out of cancer cells, thereby reducing their efficacy [106,124]. MT has been shown to reverse drug resistance in MCF-7 cells by regulating the expression of drug-resistant genes and inhibiting the function of drug-resistant proteins such as P-gp and MRP1. The phenomenon of multidrug resistance may also be intricately linked to the PI3K/AKT-signaling pathway. In their 2005 study, Kim et al. [125] discovered that activation of the PI3K/AKT-signaling pathway not only stimulated the proliferation and differentiation of breast cancer cells but also inhibited cell apoptosis, consequently bolstering their resistance to chemotherapeutic agents [126,127,128,129,130]. Activation of the PI3K/AKT pathway rendered breast tumor cells resistant to cisplatin, paclitaxel, and pirarubicin chemotherapy drugs [131,132,133,134,135]. Upon exposure of breast cancer cells to chemotherapeutic agents, AKT phosphorylation levels increase, activating the PI3K/AKT-signal pathway. Activated AKT subsequently triggers downstream factors that make breast cancer cells resistant to chemotherapeutic agents, ultimately leading to treatment failure. In a comparative experiment involving the AKT channel inhibitor MK2206, the potent impact of MT on PTEN within the PI3K/AKT pathway, as compared to the MK2206 positive control, suggests that MT may function as a natural AKT channel inhibitor. Furthermore, MT demonstrated a dose-dependent reduction in the expression of MDR1 gene products P-gp and MRP1 protein. Western blot analysis revealed that increasing concentrations of MT were associated with elevated levels of PTEN protein expression, leading to inhibition of AKT activation. This finding is consistent with the observed gradual decrease in p-AKT expression levels with escalating concentrations of MT. MT was found to inhibit this pathway, reduce P-gp and MRP1 expression, and enhance the expression of PTEN, a tumor suppressor that counteracts AKT activation. These findings suggest that MT may be a promising natural compound to overcome drug resistance in breast cancer, offering a new therapeutic avenue for resistant tumors [62].

## 5. Medications and Combination Therapies of MT

MT, a major bioactive alkaloid extracted from *S. flavescens*, has been employed in clinical practice in China since 1995. Formulations such as CKI, which contains both MT and oxymatrine, are approved by the National Medical Products Administration (NMPA) and widely used as adjuvant therapies for managing symptoms associated with various malignancies, including breast cancer, hepatocellular carcinoma, and lung cancer [35,36,136]. These formulations are primarily utilized for palliative purposes, such as alleviating cancer-related pain and controlling hemorrhage. Topical MT-based products, such as Compound Kushen Lotion and MT Lotion, have also been introduced in dermatological and gynecological indications, including eczema and vaginitis, where they exert anti-inflammatory and antimicrobial effects [137,138].

In contrast, preclinical studies have consistently demonstrated that MT may enhance the therapeutic efficacy of conventional anticancer agents. For example, in Bcap-37 cells, the combination of MT with tamoxifen increased apoptosis by 62% compared to tamoxifen alone (*p* < 0.01), mediated by ERα/BCL-2-signaling modulation [74]. In MCF-7 cells, co-treatment with MT and doxorubicin resulted in a 78% reduction in cell viability versus 45% with monotherapies, along with a substantial shift in IC_50_ values (from 1.2 μM to 0.4 μM) [56]. In triple-negative MDA-MB-231 cells, MT enhanced docetaxel’s anti-metastatic effect through downregulation of HN1 (↓82%) and VEGF/CD31 expression (↓67%) [65]. Pirarubicin–cisplatin combination therapy showed 42% higher tumor inhibition when administered with MT (*p* < 0.001), attributed to reduced ABC transporter activity [62]. Despite their clinical availability, it is important to note that rigorous randomized clinical trials evaluating MT or CKI in combination with standard chemotherapeutics in breast cancer are currently lacking. Their application in oncology remains largely empirical and based on traditional use or observational data. The efficacy and safety of these combination strategies have not been systematically validated in human populations.

These findings support the potential use of MT as a chemosensitizing and adjuvant compound, particularly in drug-resistant or metastatic phenotypes. Currently, no clinical studies have directly compared MT monotherapy with combination therapy (e.g., MT + tamoxifen) in breast cancer patients. The promising synergy observed in preclinical studies underscores the need for well-controlled, multi-arm clinical trials to assess treatment efficacy, safety, and optimal combinations.

Due to its multi-targeted pharmacological actions, including modulation of apoptosis, angiogenesis inhibition, and reversal of chemoresistance, MT presents a promising adjuvant candidate in breast cancer therapy. However, its clinical application should be restricted to adjuvant use until high-quality human data become available.

## 6. Pharmacokinetics and Toxicological Profile of MT

### 6.1. Pharmacokinetics and Dose Optimization

Understanding the pharmacokinetics (PK) of MT is essential for guiding rational dose selection and optimizing its therapeutic application in oncology. Preclinical studies in rats have shown that MT is rapidly absorbed following oral administration, with a peak plasma concentration typically reached within 0.5 to 1.5 h and an elimination half-life ranging from 1.9 to 3.2 h [139,140]. MT distributes broadly to the liver, kidneys, and lungs and is primarily eliminated via hepatic metabolism and renal excretion. However, the oral bioavailability of MT is limited due to first-pass hepatic metabolism, and maintaining therapeutic concentrations may require frequent dosing or alternative delivery routes. Research has found that the plasma concentration of MT after transdermal administration is higher than that after intravenous administration [141]. Due to the short half-life and modest bioavailability, dose optimization for anticancer purposes will require PK–PD modeling, ideally supported by clinical data from cancer patients, to ensure adequate tumor exposure without inducing toxicity.

### 6.2. Toxicological Profile and Reported Adverse Effects

Although MT is generally considered safe at clinical doses, multiple studies have reported dose-dependent toxicological effects, particularly when administered at high doses or over extended periods.

In an acute toxicity assay, Yang et al. [142] determined the median lethal dose (LD_50_) of MT in mice to be 570.26 mg/kg, indicating moderate acute toxicity. Gong et al. [143] found that MT concentrations above 140 mg/L induced hepatotoxicity in hepatocytes after 72 h, evidenced by reduced cell viability and total protein content. Chronic exposure to MT at 40 mg/kg/day for 60 days also resulted in signs of neurotoxicity in mice [144].

Biochemical analysis by Gu et al. [145] revealed elevated serum ALT and AST levels after MT administration, suggesting hepatic stress. Zebrafish studies further confirmed MT-induced liver injury, potentially mediated by oxidative stress, as shown by altered levels of malondialdehyde (MDA) and glutathione (GSH) [146]. Similarly, Li et al. [147] and others reported that inflammation and redox imbalance contribute to MT-induced hepatotoxicity. In HL-7702 cells, MT treatment resulted in a time- and concentration-dependent reduction of superoxide dismutase (SOD) and GSH levels, supporting this mechanism [148].

In terms of reproductive safety, Luo et al. [149] found that MT at concentrations ≥ 100 µM impaired sperm function in mice following only 2 h of exposure in the cauda epididymis, raising concerns about potential reproductive toxicity.

Taken together, these findings suggest that MT’s adverse effects are closely related to both dose and duration of exposure, with the liver, nervous system, and reproductive organs being particularly vulnerable. While clinical use remains relatively safe within approved indications and doses, rigorous toxicity profiling and close monitoring will be necessary in the context of long-term or high-dose anticancer applications.

## 7. Summary and Prospect

MT is a bioactive alkaloid that has exhibited robust anti-breast cancer activity in both in vitro and in vivo preclinical models. This review provides a comprehensive evaluation of MT’s effects on key cellular processes, including proliferation, viability, apoptosis, autophagy, and migration, across various breast cancer subtypes. We have summarized how MT modulates the expression of multiple genes and proteins, emphasizing the importance of concentration, dosage, and treatment duration across different cell lines. The current literature indicates that MT exerts strong dose- and time-dependent cytotoxicity, with MCF-7 cells being particularly sensitive (IC_50_ = 0.7–0.9 mg/mL), suggesting potential subtype-specific responsiveness.

The molecular mechanisms underlying MT’s anti-tumor effects vary with the breast cancer subtype. In ER+/PR+ cell lines, MT has been shown to reduce mitochondrial membrane potential, arrest the cell cycle, and inhibit telomerase activity. In TNBC models, MT primarily acts through the PI3K/Akt, MAPK/ERK, and NF-κB pathways, leading to autophagy activation, enhanced apoptosis, and reduced migration. In HER2+ cells, MT decreases miR-21 levels and concurrently upregulates PTEN and ANXA3, highlighting its pleiotropic and subtype-dependent activity. Beyond direct effects on proliferation and survival, MT regulates multiple oncogenic and tumor-suppressor pathways, including Let-7, c-Myc, Ras, Wnt/β-catenin, and JAK/STAT3, thereby halting tumor growth and cell cycle progression. MT also modulates apoptosis-related proteins (e.g., Bax, Bcl-2), inhibits telomerase activity, and promotes ANXA3 expression, all of which contribute to tumor suppression. It activates autophagy via the AKT/mTOR axis (↑LC3-II, ↓p62), inhibits EMT-related proteins, and impedes metastasis. Furthermore, MT downregulates VEGF and Wnt/β-catenin signaling, thereby impairing angiogenesis. Importantly, MT enhances immune modulation and has been reported to reverse multidrug resistance by inhibiting P-gp and MRP1, increasing PTEN levels, and reducing p-AKT activation.

Despite these encouraging findings, several gaps remain. First, the majority of studies focus solely on tumor cell lines, with limited data available on non-tumor breast epithelial cells, which hinders assessment of MT’s selectivity and safety profile. Future investigations should include comparisons between cancerous and non-cancerous cells to better understand therapeutic windows. Second, although multiple subtypes of breast cancer cells (e.g., MCF-7, T47-D, MDA-MB-231, SK-BR-3, and 4T1) have been studied, comparative analyses between subtypes are largely lacking, and the findings have not been extended to clinical trials. Third, we emphasize that all such findings are limited to in vitro and in vivo animal models. The translational gap between these controlled laboratory conditions and complex clinical scenarios, such as interpatient variability, pharmacokinetics, immune modulation, and tumor heterogeneity, must be carefully considered. As such, the anticancer activity observed in cell lines should not be assumed to reflect clinical efficacy without further clinical validation. Moreover, MT’s utility as an adjuvant to standard chemotherapy warrants further exploration. Some preclinical studies suggest synergistic effects when MT is combined with agents like tamoxifen, doxorubicin, and docetaxel, but no clinical trials have been conducted to evaluate these combinations in patients with breast cancer. Finally, MT exhibits poor water solubility and limited oral bioavailability, restricting its clinical applicability. Future studies should prioritize the development of enhanced drug-delivery systems (e.g., nanoparticles, liposomes) to improve MT pharmacokinetics and the therapeutic index.

In summary, MT exhibits significant preclinical promise for the treatment of breast cancer. However, its full therapeutic potential will depend on resolving key challenges, including mechanistic elucidation across subtypes, in vivo validation, pharmacokinetic optimization, and rigorous clinical evaluation. This review underscores the need for further comparative and translational research, particularly in ER+, PR+, HER2+, and TNBC subtypes, to support MT’s integration into future breast cancer therapeutic strategies.

## Figures and Tables

**Table 1:** The natural sources of matrine.

Familia	Genus	Name	Part Used	Reference
Leguminosae	*Sophora* L.	*Sophora flavescens* Aiton	Roots	Yang et al., 2024 [15]
Leguminosae	*Sophora* L.	*Sophora alopecuroides* L.	Fruits	Chen et al., 2023 [16]
Leguminosae	*Sophora* L.	*Sophora xanthoantha* C. Y. Ma	Flowers, buds	Chi ZL, 2011 [17]
Leguminosae	*Sophora* L.	*Sophora davidii Kom. ex Pavol.*	Stem, leaf, flower, seed, roots	Xu et al., 2023 [18]
Leguminosae	*Sophora* L.	*Sophora mollis* Graham.	Roots	Peng et al., 2010 [19]
Leguminosae	*Sophora* L.	*Sophora pachycarpa* Schrenk ex C. A. Mey	Roots	Zheng et al., 1996 [20]
Leguminosae	*Sophora* L.	*Sophora dunnii* Prain	Roots	Ling et al., 2000 [21]
Leguminosae	*Sophora* L.	*Sophora moorcroftiana* (Benth.) Baker	Seeds	Zhao et al., 2020 [22]
Leguminosae	*Sophora* L.	*Sophora tonkinensis* Gagnep.	Seeds	Zou et al., 2023 [23]
Leguminosae	*Styphnolobium* Schott	*Styphnolobium japonicum* (L.) Schott	Flowers, leaves, branches, roots, fruits	Wang et al., 2018 [24]
Leguminosae	*Euchresta* J. Benn.	*Euchresta japonica* Benth. ex Oliv.	Roots	Yuan et al., 2023 [25]
Leguminosae	*Euchresta* J. Benn.	*Euchresta formosana* (Hayata) Ohwi	Roots	Li et al., 2014 [26]
Leguminosae	*Euchresta* J. Benn.	*Euchresta horsfieldii* (Lesch.) J. Benn.)	Roots	Li et al., 2014 [26]
Leguminosae	*Euchresta* J. Benn.	*Euchresta tubulosa* Dunn	Roots	Li et al., 2014 [26]
*Solanaceae* Juss.	*Lycium* L.	*Lycium chinense* Miller	Fruits	Zhang et al., 2024 [27]
*Bignoniaceae* Juss.	*Incarvillea* Juss.	*Incarvillea sinensis* Lam.	Grass	Song et al., 2023 [28]

**Table 2:** Effect of matrine on breast cancer cells.

Cells Type	Composition (mg·mL ^−1^)	Time of Administration (h)	Cell Proliferation Inhibition Rate/ Cell Apoptosis	Influenced Gene/ Protein	Other	Ref.
MCF-7	MT	48	Cell proliferation was detected by MTT assay, and the IC50 value was 0.68 ± 2.00 mg·mL^−1^.	NA	NA	Liu et al., 2008 [50]
MCF-7	MT	24, 48, 72	Cell proliferation was detected by MTT assay, and the IC50 value was 0.93 ± 9.03 mg·mL^−1^.	NA	NA	Mao et al., 2019 [51]
MCF-7	MT	48	The cell survival rate detected by the MTT method with 4.0 mg·mL^−1^ MT was 43.13 ± 5.37%.	Significant decrease in p-JAK2, p-STAT3 protein levels.	NA	Wang et al., 2023 [52]
MCF-7	MT	48	The cell survival rate detected by MTT assay, and the IC50 value was 0.85 mg·mL^−1^.	LC3-I/LC3-II levels were significantly increased, and Beclin-1 protein expression was increased.	NA	Jia et al., 2023 [53]
MCF-7	MT	NA	Cell proliferation was detected by MTT assay, and the IC50 value was 15.8 mg·mL^−1^.	NA	NA	Sun et al., 2017 [54]
MCF-7	MT	72	Cell proliferation was detected by MTT assay, and the IC50 value was 4.83 mg·mL^−1^. Cycle retention in the G1 phase, S phase, and G2/M phase cell numbers decreased.	The expression of *p21* and *p27* was upregulated, *p53* and cyclin-D1 were downregulated, and the accumulation of LC3-II was increased.	NA	Ou, 2014 [55]
MCF-7	MT	48	Cell proliferation was detected by MTT assay, and the IC50 value was 2.00 ± 0.17 mg·mL^−1,^ and it showed concentration-dependent inhibition.	Bax protein expression was upregulated.	NA	Zhao et al., 2018 [56]
MCF-7	MT	24, 48, 72	The inhibition rate at 72 h of action time with 4.0 mg·mL^−1^ MT was 73.29 ± 4.07%, and it showed concentration-dependent inhibition. Cell proliferation was stagnant in the G0/G1 phase, and the number of cells in S phase was reduced.	Bax protein expression was upregulated and downregulated Bcl-2 protein expression.	NA	Sui, 2013 [57]
MCF-7	MT	24, 48, 72	The inhibition rate at 72 h of action time with 2.0 mg·mL^−1^ MT was 68. 31 ± 2.13% and it showed concentration-dependent inhibition and promoted apoptosis.	Decreased mitochondrial transmembrane potential and increased Bax expression.	NA	Wang et al., 2012 [58]
MCF-7	MT	24	The inhibition rate at 24 h of action time with 2.0 mg·mL^−1^ MT was 19.63 ± 0.17%.	Decreased mitochondrial transmembrane potential.	Cells show shortened protrusions, reduced cytoplasm, intracytoplasmic vacuoles, and nuclear consolidation and fragmentation.	Li et al., 2011 [59]
MCF-7	MT	24, 48, 72	The inhibition rate at 72 h of action time with 2.0 mg·mL^−1^ MT was 67.16 ± 2.14%, and it showed concentration-dependent inhibition. The apoptosis rate of 2.0 mg·mL^−1^ MT for 24 h was 20.16 ± 0.16%.	Decreased mitochondrial transmembrane potential	NA	Gu et al., 2015 [60]
MCF-7	MT	24	Significantly inhibited growth and promoted apoptosis.	Upregulation of Fas protein expression and downregulation of VEGF protein and telomerase activity gradually decreased with increasing concentrations of MT.	Cells show shortened protrusions, reduced cytoplasm, and nuclear consolidation and fragmentation with a budding of the cell membrane.	Li et al., 2013 [61]
MCF-7	MT	24	The MTT assay showed a concentration- and time-dependent inhibition rate of 22.35 ± 0.84% for mg·mL^−1^ MT at 24 h.	Decreased p-pg, MRP1 protein, and p-AKT protein expression in the PI3K/AKT-signaling pathway and increased PTEN protein expression.	NA	Wei et al., 2016 [62]
MCF-7	MT	72	The inhibition rate of MCF-7/ADR cells at 1.25 mg·mL^−1^ MT was 66.2% with an IC50 of 0.92 mg·mL^−1^. The proportion of S-phase cells increased, and the proportion of G2/M cells decreased.	NA	Cytosol shrinkage and cytoplasmic condensation.	Zhou et al., 2003 [63]
MCF-7	MT	24	After 24 h, the concentrations of 2.0 mg·mL^−1^ and 2.4 mg·mL^−1^ of MT induced the late apoptosis rate to be 23.5% ± 0.024 and 56.82% ± 0.042. The IC50 at 24 h was 2.729 mg·mL^−1^.	Inhibits the expression of IL-6, JAK1, P-JAK1, STAT3, and P-STAT3 proteins by a molecular mechanism that may be related to its effective regulation of the IL-6/STAT3 pathway.	NA	Ren, 2023 [64]
MDA-MB-231	MT	24, 48, 72	The inhibition rate at 72 h of action time with 5 mg·mL^−1^ MT was 67.41%.	Downregulation of HN1 protein expression and decreased expression of VEGF and CD31.	NA	Guo, 2022 [65]
Bcap-37	MT	48	The inhibition rate at 48 h of action time with 2.0 mg·mL^−1^ MT was 18.25 ± 1.12%.	Decreased Cyclin D1 and c-Myc protein expression.	NA	Xiao et al., 2018 [66]
4T1	MT	48	The inhibition rate at 48 h of action time with 0.4 mg·mL^−1^ MT was 17.32 ± 3.09%.	Significantly decreased ANXA3 protein expression.	NA	Shi et al., 2018 [67]
SK-BR-3	MT	48	The inhibition rate at 48 h of action time with 3.0 mg·mL^−1^ MT was 46.6 ± 3.2% and showed concentration-dependent inhibition.	Inhibition of *miR-21* expression and upregulation of PTEN protein expression.	NA	Xiao et al., 2018 [68]
MCF-7	MT	72	Cell proliferation was detected by MTT assay, and the IC50 value was 0.92 mg·mL^−1^.	The relative expression of Bcl-2 and *p65* was decreased, and the relative expression of Bax, PARP, and *GSK-3β* genes was increased.	NA	Zhou et al., 2017 [69]
Bcap-37	MT	24, 48, 72	Cell proliferation was detected by MTT assay, proliferation inhibition existed in a concentration and time-dependent manner, and the apoptosis rate was 19.58% at 24 h of the action time of 2 mg·mL^−1^.	LC3b-II expression was upregulated.	NA	Ren et al., 2014 [70]
MDA-MB-231	MT	48	MTT assay was used to detect cell proliferation, and the inhibition rate was 49.21 ± 1.12% at 0.2 mg·mL^−1^ action time 48 h.	It may be related to PI3K/Akt and MAPK/ERK-signaling pathway.	NA	Yu et al., 2016 [71]
MCF-7/ADR cell	MT	24	The MTT assay showed a concentration- and time-dependent inhibition rate of 11.82 ± 0.66% for 0.6 mg·mL^−1^ at 24 h.	Decreased expression of MDR1, MRP1, and AKT gene and increased expression of PTEN gene.	NA	Wei et al., 2014 [72]
Bcap-37	MT	24, 48, 72	Cell proliferation was detected by MTT assay, and the proliferation inhibition was concentration- and time-dependent with IC50s of 0.63, 0.42, and 0.29 mg·mL^−1^ at 24, 48, and 72 h.	The expression of caspase-3 and Bax proteins was increased, and the expression of Bcl-2 protein was decreased.	NA	Zheng et al., 2012 [73]
Bcap-37	MT	24, 48, 72	The highest apoptosis rate of 6.90 ± 0.19% was observed when the action time of 0.08 mg·mL^−1^ was 48 h.	The expression of *p53* and Bax proteins was increased, and Bcl-2 protein was decreased.	NA	Zheng et al., 2012 [74]
MCF-7	MT	24, 48, 72	Cell proliferation was detected by MTT assay, and proliferation inhibition was concentration- and time-dependent, with inhibition rates ranging from 10.86% to 70.23% at 72 h.	Bax protein expression was increased, and Bc-l2 protein was decreased.	NA	Li et al., 2011 [75]
Bcap-37	MT	24, 48, 72	Cell proliferation was detected by MTT assay, and proliferation inhibition was concentration- and time-dependent.	Increased expression of *p53* and Bax proteins and decreased expression of Bcl-2 proteins.	Decreased cytoplasm, rounding, and vacuolization of cells.	Zheng et al., 2012 [76]
MCF-7/ADR cell	MT	48	Cell proliferation was detected by MTT assay, and proliferation inhibition was concentration- and time-dependent.	Increased expression of Fas and bax proteins and decreased Bcl-2 proteins.	NA	Zhou et al., 2007 [77]
MCF-7	MT	24	Cellular autophagy phenomenon was obvious and concentration-dependent.	The expression of Beclin-1 was increased, and PI3K, Akt, and TOR were decreased.	NA	Jia et al., 2023 [78]
MCF-7	MT	24	MTT results showed that the IC50 of MT for MCF-7 was 1.38 ± 0.09 mg·mL^−1^.	The expression of P-gp was decreased.	NA	Li et al., 2013 [79]
Bcap	MT	72	The MTT assay showed that the inhibition rate of 0.1 mg·mL^−1^ was less than 40% for 72 h.	NA	NA	Wang et al., 1996 [80]
MCF-7	MT	24, 48, 72	CCK-8 test showed MT inhibited cancer cell proliferation in a time-dose-dependent manner, and the apoptosis rate was 72.81±3.83% at 8 mg·mL^−1^ of MT.	Reduced *p62* expression and p-AKT/AKT ratio.	Cells showed contraction, membrane blistering, balloon-like protrusions, and partial detachment.	Du et al., 2020 [81]
MCF-7 BT-474 MDA-MB-231	MT	48	3 mg·mL^−1^ MT resulted in a reduction of cell number to 15.5–23.6% by MTT assay, and MDA-MB-231 was the most sensitive cell. 3 mg·mL^−1^ MT acted for 48 h, and the apoptosis rate was about 90%.	Reduced IKKβ expression, which may be related to the NF-κB-signaling pathway.	NA	Shao et al., 2018 [82]
MCF-7	MT	24, 48, 72	MTT assay showed that it inhibited cell proliferation in a concentration- and time-dependent manner and induced apoptosis with an IC50 value of 0.86 mg·mL^−1^ at 48 h.	Inhibited the expression of vascular endothelial growth factor and down-regulated the Wnt/β-catenin pathway.	NA	Xiao et al., 2018 [83]
MCF-7	MT	24, 48, 72	MTT assay showed that MT inhibited cell growth in a concentration- and time-dependent manner with an IC50 (48 h) of approximately 0.8 mg·mL^−1^. After the action of MT with 0.8 mg·mL^−1^, the apoptosis rate was 25.6 ± 4.8%, inducing cell cycle arrest in G1/S phase.	Increased expression of PTEN, *p21*/WAF1/CIP1, *p27*/KIP1 and decreased expression of pAkt, pBad	NA	Li et al., 2019 [84]
MCF-7 T47-D	MT	24, 48, 72	MTT assay showed that MT inhibited cell growth in a concentration- and time-dependent manner with an IC50 (48 h) of approximately 0.8 mg·mL^−1^.	Increased expression of Let-7b and decreased expression of CD133, *KLF4.*	NA	Liang et al., 2019 [85]
MDA-MB-231 MCF-7	MT	24, 48	MTT assay showed that MT inhibited cell growth in a concentration- and time-dependent manner and induced apoptosis.	Increased expression of E-calmodulin and decreased expression of N-calmodulin and waveform protein.	MT(2 mg/mL) significantly inhibited cell migration.	Ren et al., 2020 [86]
MCF-7	MT	48	MTT assay showed MT significantly inhibited cell proliferation and induced cell apoptosis. S-phase cells decreased and G0/G1-phase cells increased.	Bax protein expression increased and Bcl-2 protein expression decreased.	NA	Shi et al., 2015 [87]
MDA-MB-453 HCC-1806	MT	NA	CCK-8 tests indicate that MT inhibits cell proliferation and promotes apoptosis.	HN1 protein expression is inhibited and Cleared-Caspase-3 protein expression is increased.	NA	Guo et al., 2023 [88]
MCF-7	MT	24, 48, 72	Cell viability was detected by MTT assay with an IC50 value of 0.53 ± 34.6 mg·mL^−1^.MT promoted apoptosis.	Promoted the release of cytochrome C and enhances caspase-3 activity, while *p-eIF2a* was elevated.	NA	Xiao et al., 2017 [89]
MCF-7	MT	24, 48, 72	MTT assay showed that the inhibition of cell proliferation after 72 h was 10.86–70.23% in a time- and concentration-dependent manner, and the apoptosis rate was 4.17–19.63% in the 0.25–2.0 mg·mL^−1^ MT group.	Increased expression of Bax and decreased expression of Bcl-2.	NA	Li et al., 2015 [90]
BT-20 MCF-7	MT	48	The IC50 values of cell viability after 48 h were 0.31 ± 23.45 mg·mL^−1^ and 0.22 ± 16.89 mg·mL^−1^ as detected by the MTT assay.	NA	NA	Jiang et al., 2017 [91]
MCF-7	MT	24, 48, 72	Cell survival was 77.5%, and apoptosis was 32.6% as detected by flow cytometry.	NA	NA	Zhang et al., 2016 [92]
MDA-MB-231 MDA-MB-468	MT	24, 48, 72	MT inhibited cell proliferation, and flow cytometry results showed that MT blocked the cell cycle and induced apoptosis.	Inhibition of BCL-2 expression and up-regulation of caspase-3, LC3-II expression.	NA	Wei et al., 2023 [93]
MCF-7	MT	24, 48, 72	MTT assay showed that MT inhibited cell growth in a concentration- and time-dependent manner and induced apoptosis.	Decreased Bcl-2/Bax ratio, downregulated VEGF and VEGFR-2 expression, and increased caspase-3 and caspase-9.	NA	Li et al., 2010 [94]
MCF-7 MDA-MB-231	MT	8, 16, 24	MTT assay showed that MT inhibited cell growth in a concentration- and time-dependent manner.	Increased expression of Akt, Erk1/2, and *p38*.	NA	Zou et al., 2019 [95]
MDA-MB-231	MT	NA	The inhibition rate of cell proliferation after 0.1 mg·mL^−1^ of MT was 20.29 ± 0.84% as determined by MTT assay.	NA	NA	Thang PNT et al., 2022 [96]
MCF-7	MT	48	The MTT assay showed MT inhibited cell viability in a concentration-dependent manner, and the IC50 value for cell viability after 48 h was 0.53 mg·mL^−1^.	NA	NA	Mousavi SH et al., 2014 [97]
MDA-MB-231	MT	24, 48, 72	MTT assay showed that MT inhibited cell growth in a concentration- and time-dependent manner.	Decreased Bcl-2/Bax protein and decreased mRNA levels of MMP-9, MMP-2, EGF, and VEGFR1.	NA	Yu et al., 2009 [98]
MDA-MB-231	MT	24	MT inhibited cell growth and induced apoptosis.	Increased caspase-3 activation.	NA	Chui CH et al., 2005 [99]

**Table 3:** Anti-breast cancer effect of matrine in vivo.

Cells Type	Composition/Concentration	Dosage	Time Administer Drug	Methods	Results	Ref.
4T1	MT, 3.5–7.5 mg·mL^−1^	10 mL·kg^−1^	10	Mice in the modeling group were inoculated with 0.2 mL of 1 × 10^6^ cells-mL^−1^ of single-cell suspension of mouse 4T1 breast cancer cells in the right axilla, and after successful modeling, mice were injected with MT once/d for 10 d.	The tumor weight of mice in groups of MT was reduced, and the number of apoptotic cells was higher. The effect may be related to the activation of the JNK1/AP-1-signaling pathway and the regulation of *p53,* Bax, and other apoptosis-related proteins.	Dong, 2019 [100]
MDA-MB-231	MT, 20 mg·mL^−1^	50 mL·kg^−1^	15	The nude mice were inoculated with 1 × 10^7^ cells-mL^−1^ of MDA-MB-231 cell suspension under the right mammary gland, and MT 50 mg/kg was injected intraperitoneally five times/week for a total of 15 times.	Tumor growth was inhibited in nude mice after the administration of MT. MT can induce apoptosis. At day 22, the inhibition of tumor volume by ascorbic acid alone was 28.72%.	Guo et al., 2022 [65]
Walker 256	MT, 0.5–20 mg·mL^−1^	10 mL·kg^−1^	28	Rats were subcutaneously inoculated with 0.4 mL of Walker 256 breast cancer cell suspension (5 × 10^7^ cells-mL^−1^) in the right axilla. The drug was administered intraperitoneally at a volume of 10 mL·kg^−1^ twice/day for 14 d.	The tumor inhibition rates of the low-, medium-, and high-dose groups of MT were 24.6%, 31.7%, and 36.3%, respectively, showing a dose-dependent inhibition of tumor growth.	Zhang et al., 2018 [101]
MA737	MT, NA	20 mL·kg^−1^	28	MA737 cells 1 × 10^5^ cells/mouse were inoculated subcutaneously in the right inguinal area, randomly grouped, and sacrificed 14 days after administration, and thymus, spleen, and tumor weights, as well as body weights of the mice, were weighed.	MT can increase the weight of the thymus and spleen, and the weight of the tumor is smaller. After the treatment of MT, the TH/TS ratio of mice is significantly increased, which can improve the immunity of mice.	Mao et al., 1996 [102]
MDA-MB-453	MT, NA	NA	NA	NA	MT inhibited tumor growth and decreased the expression of HN1 protein but promoted the protein expression of Cleared-Caspase-3.	Guo Q et al., 2023 [88]
4T1	MT, NA	NA	14	Injected 4 × 10^4^ 4T1 cells into the left inguinal mammary fat pads of mice, after 15 days, received daily i.p. injection of MT for 14 days, and then euthanized, and the tumors, lungs, and livers were removed.	The volume of tumors of mice treated with MT was significantly smaller, and the numbers of tumor on the lung and livers surface of MT-treated mice were lowered.	Li H et al., 2010 [94]
4T1	MT, NA	NA	21	Injected 4 × 10^4^ 4T1 cells (100 µL, 5 × 10^6^ cells-mL^−1^) into the right side of the fourth mammary gland of mice, mice received i.p. injection of MT, the mice were sacrificed on day 21, and the tumors were removed rapidly and weighed.	MT can inhibit the growth of tumors and induce apoptosis while decreasing the level of VEGF.	Xiao X et al., 2018 [83]
TM40D	MT, 5–20 mg·mL^−1^	10 mL·kg^−1^	20	TM40D cells with cell density adjusted to 2.0 × 10^7^ cells-mL^−1^, subcutaneous injection of 0.1 mL per nude mouse’s back, and intraperitoneal injection of the corresponding dose of drug. On the 20th day, the mice were euthanized, and tumor blocks were removed and weighed.	MT can reduce tumor weight and promote cancer cell apoptosis.	Bai et al., 2016 [103]
MCF-7/ADR	MT, 50, 100 mg·mL^−1^	NA	21	Nude mice were subcutaneously inoculated with MCF-7/ADR cells. MT was injected for 21 days, and the tumor inhibition rate was measured using in vivo tumor inhibition experiments.	The tumor inhibition rates of the high-dose and low-dose groups of MT were 60.7% and 42.1%. MT can promote cancer cell apoptosis.	Zhou et al., 2017 [104]

## Abbreviations

The following abbreviations are used in this manuscript:

ANXA3, annexin A3; Bax, Bcl-2-associated X protein; Bcl-2, B-cell lymphoma 2; BCSC, breast cancer stem cells; BP, biological process; CC, cellular component; CKI, the compound Kushen injection, *E. japonica, Euchresta japonica* Benth. ex Oliv. EMT epithelial-mesenchymal transition; ER+, Estrogen receptor (+); GO, gene ontology; HER2+, Human Epidermal Growth Factor Receptor 2 (+); IARC, International Agency for Research on Cancer; JAK-STAT, Janus kinase-signal transducer and activator of transcription; KEGG, kyoto encyclopedia of genes and genomes; IL-17, Interleukin 17; MAPK/ERK, mitogen-activated protein kinase/extracellular regulated protein kinases; MT, matrine; MMP, mitochondrial membrane potential; MRP1, Multidrug Resistance-Associated Protein 1; mTOR, mammalian target of rapamycin; NF-κB, nuclear factor kappa-B; PI3K-Akt, Phosphoinositide 3-kinase-Akt; PK, pharmacokinetics; PR+, progesterone receptor (+); PTEN, Phosphatase and Tensin Homolog; P-gp, P-glycoprotein; *S. flavescens, Sophora flavescens* Aiton; *S. alopecuroides, Sophora alopecuroides* L.; TCM, Traditional Chinese Medicine; TNBC, triple-negative breast cancer; TNF, Tumor Necrosis Factor; VEGF, vascular endothelial growth factor.
